# Impact of hyperglycemia and antidiabetic medication on pancreatic uptake on [^68^Ga]Ga-DOTATOC PET/CT

**DOI:** 10.3389/fendo.2025.1536301

**Published:** 2025-05-16

**Authors:** Sophie Carina Kunte, Thorsten Siegmund, Maximilian Tiling, Lukas Ostermair, Lena Maria Unterrainer, Marily Theodoropoulou, Martin Reincke, Friederike Völter

**Affiliations:** ^1^ Department of Nuclear Medicine, University Hospital, LMU Munich, Munich, Germany; ^2^ Bayerisches Zentrum für Krebsforschung (BZKF), Partner Site Munich, Munich, Germany; ^3^ Division for Endocrinology, Diabetology and Metabolism, Isar Clinic Munich, Munich, Germany; ^4^ Department of Medicine IV, Endocrinology, Diabetes and Metabolism LMU University Hospital, LMU Munich, Munich, Germany; ^5^ Ahmanson Translational Theranostics Division, David Geffen School of Medicine at UCLA, Los Angeles, CA, United States

**Keywords:** glucose, diabetes, pancreas, HbA1c, SSTR PET/CT, Sst, [68 Ga]Ga-DOTATOC, sensitivity

## Abstract

**Introduction:**

Positron-emission-tomography-(PET)/computed-tomography-(CT) using somatostatin-receptor-(SSTR)-binding radioligands is well established in the imaging of neuroendocrine tumors (NETs). SSTRs are expressed in NETs and endocrine and exocrine tissues, e.g. pancreas, where somatostatin binding to SST2 and SST5 inhibits glucagon and insulin secretion. Pancreatic background activity on SSTR-PET varies widely and is increased in up to 45% of cases. High uptake in the *processus uncinatus* can obscure NETs or cause false positives. The determinants of elevated pancreatic activity on SSTR-PET remain unclear, prompting investigation into the association between pancreatic radioligand uptake and diabetic status.

**Methods:**

All patients with non-pancreatic NETs undergoing [68Ga]Ga-DOTATOC-PET/CT at LMU clinic with available HbA1c were included. Patients were grouped: without glucose metabolism disorder (HbA1c 4.0-5.6%), prediabetes (HbA1c 5.7-6.4%), type 2 diabetes mellitus. Pancreatic volume and tracer uptake were assessed, with correlation and regression analyses between SSTR expression and HbA1c.

**Results:**

The study included 40 patients (54 scans; n=22: normal glucose metabolism, n=20: prediabetes, n=12: diabetes; n=11: antidiabetic medication (AM)). Patients with normal glucose homeostasis showed increased tracer-uptake than those with impaired glucose metabolism (p=0.033; p=0.009). Correlation analysis revealed a significant negative correlation of HbA1c and SUVmax in patients without AM (r2 = 0.267; p<0.001). Multiple linear regression analysis with AM as a covariate revealed a significant association between HbA1c and SUVmax (r2 = 0.667; CI -0.371 to -0.135; p<0.001), AM was a significant covariate (CI 1.393 to 2.120; p<0.001). The association between HbA1c and SUVmean showed a trend (p=0.061) but no statistical significance.

**Conclusion:**

Our findings indicate a significant association between pancreatic [68Ga]Ga-DOTATOC-uptake and glucose metabolism, suggesting that [68Ga]Ga-DOTATOC-PET/CT sensitivity for detecting pancreatic NETs may be affected by individual glucose homeostasis.

## Introduction

Somatostatin receptors (SST) are valuable targets for *in vivo* imaging through positron emission tomography (PET) using SST-binding radioligands. SSTR-PET has become an established diagnostic tool for the detection and staging of well differentiated neuroendocrine tumors (NETs) that express SSTs (1, 2). Various radioligands used in SSTR-PET exhibit different affinities for SSTR subtypes: [^68^Ga]Ga-DOTATATE and [^18^F]SiTATE bind predominantly to SST2, [^68^Ga]Ga-DOTANOC targets SST2, 3 and 5, and [^68^Ga]Ga-DOTATOC has a high affinity for SST2 and, to a lesser extent to SST5 (3–6). Multiple organs with physiologic SST expression show an enhanced radioligand uptake on SSTR-PET, including the pituitary and the pancreas. The SSTR subtypes targeted by [^68^Ga]Ga-DOTATOC SST2 and SST5 are expressed on α- and β-cells in human islets (7) and in acinar cells of the exocrine pancreas (8–12). Additionally, SST2 is expressed in pancreatic polypeptide cells (13).

Somatostatin is a regulatory hormone of the glucose metabolism and is produced in the δ cells of the endocrine islet of Langerhans. By binding to SST2 and SST5 on pancreatic α and β cells, it inhibits glucagon and insulin secretion directly via paracrine secretion (7, 14, 15). Additionally, it acts indirectly by suppressing GLP-1 secretion from enteroendocrine L cells (14, 16). Dysregulation of these negative regulatory loops is a pathophysiologic characteristic in patients with diabetes (17, 18). Despite these insights, little is known about how diabetes, hyperglycemia and antidiabetic medication affect pancreatic SST expression and radioligand uptake on SSTR-PET.

Existing literature suggests, that diabetes or hyperglycemia can indeed alter pancreatic radioligand uptake: Sako et al. demonstrated reduced pancreatic [^68^Ga]Ga-DOTATOC uptake in rats with streptozotocin induced diabetes, which was attributed to the β cell loss (19). Similarly, Oh et al. identified a negative correlation between [^68^Ga]Ga-DOTATOC uptake in the *processus uncinatus* of the pancreas and blood glucose levels (20).

In clinical practice, increased pancreatic tracer uptake can obscure tumor detection or lead to false positive results when focal, complicating the diagnostic process. Thus, the aim of the study was to investigate the impact of hyperglycemia and antidiabetic treatment on pancreatic tracer uptake on [^68^Ga]Ga-DOTATOC PET, with the goal of enhancing diagnostic accuracy for patients with pancreatic NETs.

## Patients and methods

### Patients

We included all patients with a non-pancreatic NET (e.g. intestinal NET or carcinoid) who had undergone [^68^Ga]Ga-DOTATOC PET/CT in our department with available blood serum samples including HbA_1c_ within 2 weeks of the PET scan consecutively from 01.01.2018 until 31.12.2023. Patients with a NET of the pancreas or any pre-treatment that might have affected the pancreas were excluded. According to the regulations of the German Pharmaceuticals Act §13(2b), all patients gave written consent to undergo PET/CT. This analysis was performed in compliance with the principles of the Declaration of Helsinki and approved by the institutional ethics board of the LMU Munich (IRB #21-0102, #23-0689).

### Patient classification using HbA_1c_

The patients were classified using HbA_1c_ according to the following cut-offs: normal glucose homeostasis: HbA_1c_ 4.0 - 5.6%; impaired glucose homeostasis (prediabetes): HbA_1c_ 5.7 - 6.4%; diabetes mellitus: intake of diabetes medication and/or HbA_1c_ ≥ 6.5%. HbA_1C_ was measured by high pressure liquid chromatography.

### Radiopharmaceutical and imaging protocol

[^68^Ga]Ga-DOTATOC was prepared as previously described (21). The tracer was injected intravenously (mean 233.0 ± 42.9 MBq). PET/CT-scans were acquired at the Department of Nuclear Medicine, LMU Munich using a Siemens Biograph mCT flow or a Siemens Biograph 64 (Siemens Healthineers, Erlangen, Germany). Scans were acquired at a mean of 62.3 ± 9.9 min after tracer injection. Furosemide and iopromide were administered as previously described (2, 22). Images were reconstructed as described elsewhere (2).

### PET image analysis

**Figure 1:**
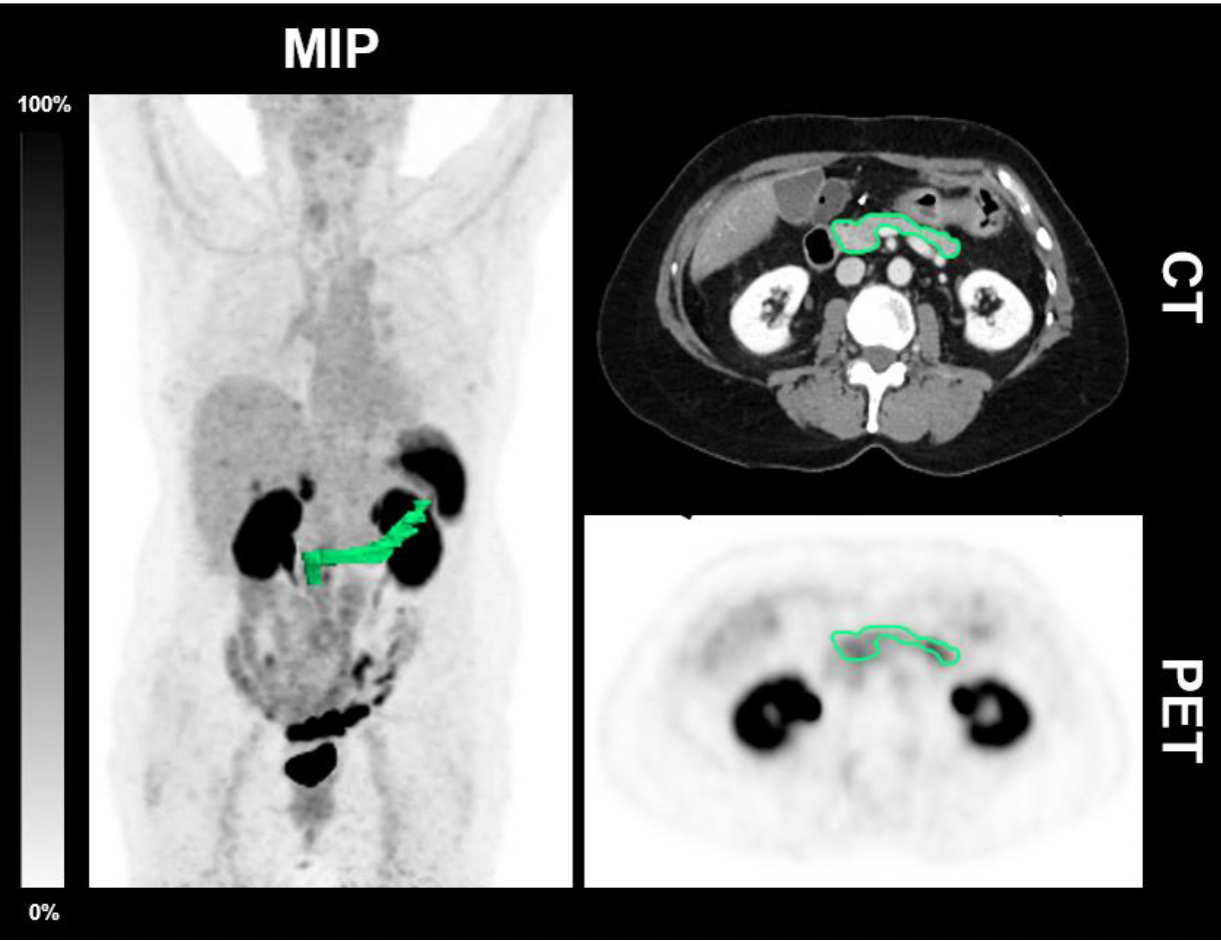
CT-derived delineation of the pancreas in a patient without T2D. Maximum intensity projection (MIP), axial CT and PET of a 57-year-old female patient diagnosed without T2D (HbA_1c_ 5.6%; 38 mmol/l) undergoing [^68^Ga]Ga-DOTATOC-PET/CT demonstrating the CT morphological delineation of the pancreas. The SUV_max_ was 5.0 and the SUV_mean_ 2.5.

A dedicated software package was used (Hermes Hybrid Viewer, Affinity 1.1.4; Hermes Medical Solutions, Stockholm, Sweden). The pancreatic volume was delineated in the CT and the standardized uptake values (SUV_max_ and SUV_mean_) of the pancreas were determined (**Figure 1**).

### Statistical analysis

Data analysis was performed using Microsoft Excel (Excel 2019, Microsoft, Redmond, WA, USA) and GraphPad Prism (Version 9.5.0 (730)). Shapiro-Wilk-normality-test was performed. Descriptive statistics are displayed as median with 1^st^ quartile (Q1) and 3^rd^ quartile (Q3) or mean ± standard deviation. Tracer uptake (SUV_mean_ and SUV_max_) was compared across all three patient groups using the Kruskal-Wallis-test. Correlation was tested using Spearman’s correlation coefficient based on normality testing. Additionally, the tracer uptake was compared to HbA_1c_ with a multiple linear regression correcting for antidiabetic medication. A two-tailed p-value < 0.05 was considered statistically significant.

## Results

### Patient characteristics

The study included 54 scans from 40 patients (21 female; 19 male) with a mean age of 65.6 ± 11.9 years (**Table 1**). Ten patients underwent two scans, two patients underwent three scans. Three patients presented with normal glucose levels at earlier scans, however, developed prediabetes over the years. 35 patients were diagnosed with a NET of the gastrointestinal system, 4 patients were diagnosed with a carcinoid of the lung and 1 patient with a NET of a tailgut cyst.

**Table 1:** Clinical characteristics of the patient cohort.

Scan no.	T2D	Sex	Age	Volume [mL]	HbA_1c_ [%]	HbA_1c_ [mmol/mol]	SUV_max_	SUV_mean_	Antidiabetic medication
1.1	no	M	59	22.4	5.7	39	5.3	3.5	
1.2	no	M	58	21.7	5.3	34	7.2	3.7	
1.3	no	F	57	19.2	5.9	41	5.8	2.3	
1.4	no	F	56	19.5	6.0	42	6.1	2.8	
1.5	no	M	70	16.1	6.0	42	4.6	2.5	
1.6	no	M	69	26.7	5.8	40	4.5	2.1	
1.7	no	M	41	31.1	5.2	33	7.0	4.0	
1.8	no	M	59	20.3	5.6	38	6.6	3.6	
1.9	no	F	58	19	5.5	37	5.9	3.7	
1.10	no	F	76	25.4	5.9	41	5.8	2.6	
1.11	no	F	62	22.3	5.7	39	4.2	1.6	
1.12	no	M	51	38.8	5.6	38	6.2	3.0	
1.13	no	M	51	39.5	5.7	39	6.9	3.6	
1.14	no	F	38	80.3	4.5	26	8.8	2.9	
1.15	no	F	87	15.8	6.0	42	7.1	4.0	
1.16	no	F	47	25.8	5.3	34	9.8	5.1	
1.17	no	M	72	36.2	5.5	37	6.2	3.0	
1.18	no	M	73	38.3	5.6	38	6.8	3.2	
1.19	no	F	49	25.7	5.3	34	6.9	2.6	
1.20	no	F	63	62.3	6.0	42	6.2	3.0	
1.21	no	F	72	15.2	5.6	38	6.3	3.1	
1.22	no	F	72	16.3	5.6	38	7.4	4.0	
1.23	no	F	86	18.9	5.5	37	8.1	3.1	
1.24	no	F	86	13.5	5.2	33	6.8	4.0	
1.25	no	M	71	23.7	5.2	33	7.2	2.8	
1.26	no	M	70	22.5	5.7	39	4.9	2.3	
1.27	no	M	69	22.6	5.0	31	8.3	2.6	
1.28	no	F	57	21.50	5.6	38	5.0	2.9	
1.29	no	F	74	22.50	5.7	39	4.8	3.0	
1.30	no	F	85	18.50	6.0	42	6.3	4.2	
1.31	no	M	71	35.40	5.5	37	5.3	2.8	
1.32	no	M	73	37.50	5.6	38	6.8	2.9	
1.33	no	M	60	28.90	4.9	30	7.0	3.1	
1.34	no	M	54	62.70	5.7	39	7.8	3.0	
1.35	no	F	58	15.70	5.9	41	5.6	2.6	
1.36	no	F	55	30.20	5.6	38	6.0	2.9	
1.37	no	M	78	25.20	5.9	41	5.2	2.0	
1.38	no	M	87	18.60	5.9	41	4.5	2.0	
1.39	no	M	82	37.80	5.2	33	6.0	3.0	
1.40	no	F	50	22.30	5.7	39	3.1	1.4	
1.41	no	F	76	27.50	5.8	40	7.5	3.0	
1.42	no	M	56	22.50	5.7	39	6.5	2.8	
2.1	yes	M	62	19.50	9.3	78	6.0	2.9	Sitagliptin, Insulin glargine
2.2	yes	M	62	19.20	9.5	80	4.1	2.0	Sitagliptin, Insulin glargine
2.3	yes	M	73	14.20	7.2	55	5.2	2.9	
2.4	yes	F	69	15.10	6.2	44	7.8	2.6	Metformin
2.5	yes	F	70	15.30	6.4	46	7.3	3.3	Metformin
2.6	yes	M	56	97.10	6.9	52	6.7	2.4	Metformin, Insulin glargine
2.7	yes	F	77	43.40	6.8	51	7.2	3.9	Metformin
2.8	yes	F	78	42.60	7.7	61	7.0	2.6	Metformin
2.9	yes	M	74	22.50	6.9	52	6.5	3.3	Sitagliptin
2.10	yes	F	68	21.20	6.7	50	7.4	3.4	Metformin
2.11	yes	M	55	12.50	6.9	52	7.5	3.3	Sitagliptin
2.12	yes	M	56	12.40	6.6	49	6.5	3.4	Sitagliptin
Mean			65.6						
SD			11.9						
Median				22.5	5.7	39	6.5	3.0	
Q1				18.9	5.5	37	5.7	2.6	
Q3				30.9	6.0	42	7.2	3.3	

SD, standard deviation; F, female; M, male; Q1 1^st^ quartile, Q3 3^rd^ quartile.

The median overall HbA_1c_ was 5.7% (Q1: 5.5%; Q3: 6.0%; IFCC: median HbA_1c_ 39 mmol/mol, Q1: 37 mmol/mol; Q3: 42 mmol/mol). The median overall pancreatic volume was 27.5 mL (Q1: 18.9 mL; Q3: 30.9 mL). The median SUV_max_ of the pancreatic tissue was 6.5 (Q1: 5.7; Q3: 7.2) and the SUV_mean_ 3.0 (Q1: 2.6; Q3: 3.3) 22/54 PET/CT images were obtained from patients (n = 18) without any glucose metabolism disorder at a mean age of 63.6 ± 13.6 years (**Table 1**). The median HbA_1c_ was 5.5% (Q1: 5.2%; Q3: 5.6%; IFCC: median HbA_1c_ 37 mmol/mol; Q1: 33 mmol/mol; Q3: 38 mmol/mol). The median pancreatic volume was 25.8 mL (Q1: 20.6 mL; Q3: 36.0 mL). The median SUV_max_ of the pancreatic tissue was 6.8 (Q1: 6.2; Q3: 7.2). and the median SUV_mean_ 3.1 (Q1: 2.9; Q3: 3.7).

20/54 scans were obtained from patients with prediabetes (n = 17) at a mean age of 66.9 ± 12.0 years (**Table 1**). The median HbA_1c_ was 5.9% (Q1: 5.7%; Q3: 5.9%; IFCC: median HbA_1c_ 40 mmol/mol; Q1: 39 mmol/mol; Q3: 41 mmol/mol). The mean pancreatic volume was 22.5 mL (Q1: 19.1 mL; Q3: 25.7 mL). The median SUV_max_ of the pancreatic tissue was 5.7 (Q1: 4.8, Q3: 6.4) and the SUV_mean_ 2.7 (Q1: 2.3, Q3: 3.0).

**Figure 2:**
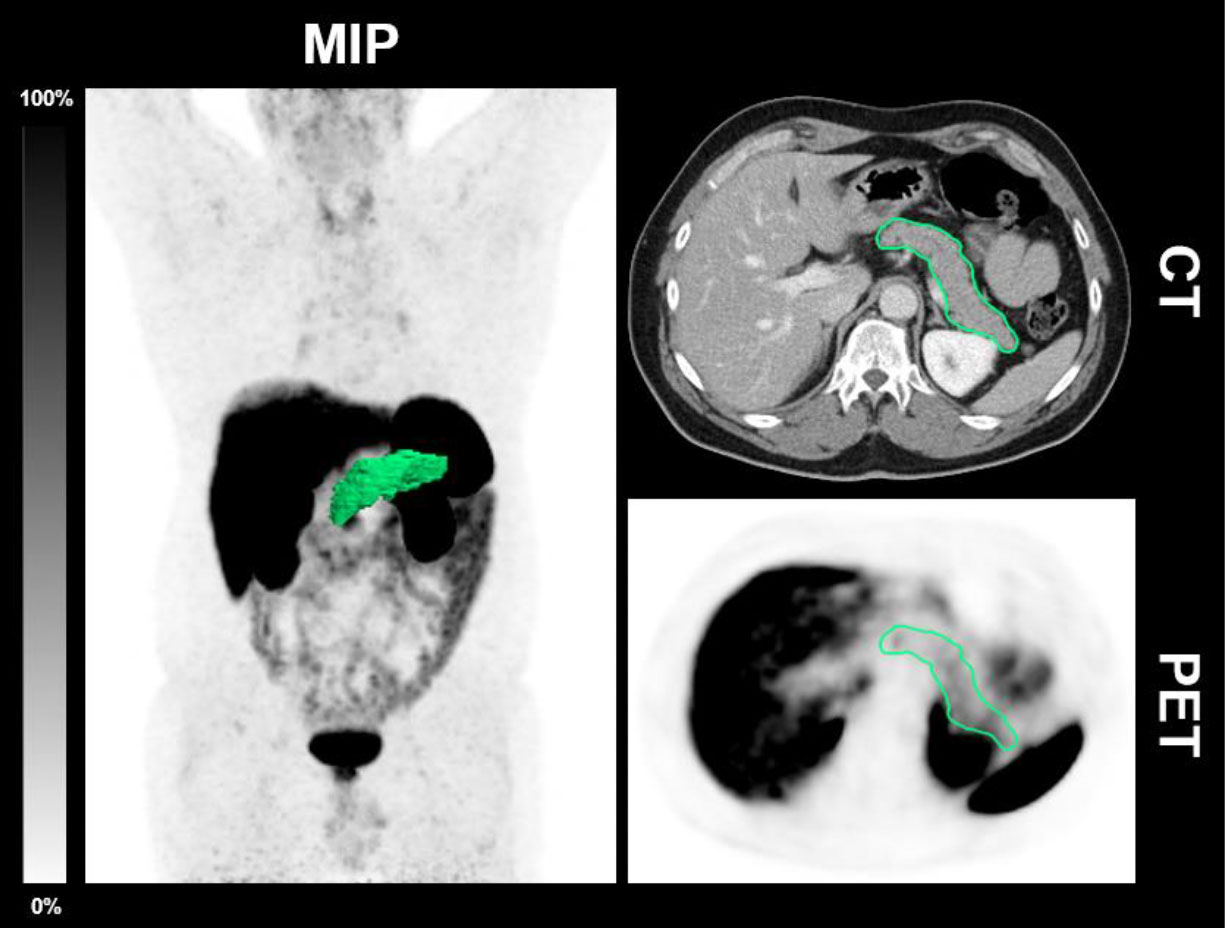
CT-derived delineation of the pancreas in a patient with T2D. Maximum intensity projection (MIP), axial CT and PET of a 56-year-old male patient diagnosed with T2D and antidiabetic medication with basal insulin supported oral therapy (Metformin and Insulin glargin) undergoing [^68^Ga]Ga-DOTATOC-PET/CT demonstrating the CT morphological delineation of the pancreas. HbA_1c_ was 6.9% (52 mmol/l); the SUV_max_ was 6.7 and the SUV_mean_ 2.4.

12/54 scans were obtained from patients (n = 8) with T2D (mean age 66.7 ± 8.3 years; **Table 1**). 11/12 scans were obtained from patients receiving antidiabetic medication. The median HbA_1c_ was 6.9% (Q1: 6.7%; Q3: 7.3%; IFCC: median HbA_1c_ 52 mmol/mol; Q1: 50 mmol/mol; Q3: 57 mmol/mol). 3/8 patients were taking metformin, 2/8 sitagliptin, 1/8 metformin and insulin glargine, 1/8 sitagliptin and insulin glargine. One patient followed dietary restrictions only. The median SUV_max_ in this cohort was 6.9 (Q1: 6.4; Q3: 7.3) and the median SUV_mean_ was 3.1 (Q1: 2.6; Q3: 3.3). The median pancreatic volume was 19.4 mL (Q1: 14.9 mL; Q3: 27.5 mL). A patient example is illustrated in **Figure 2**.

### Influence of SST tracer uptake and clinical classification

**Figure 3:**
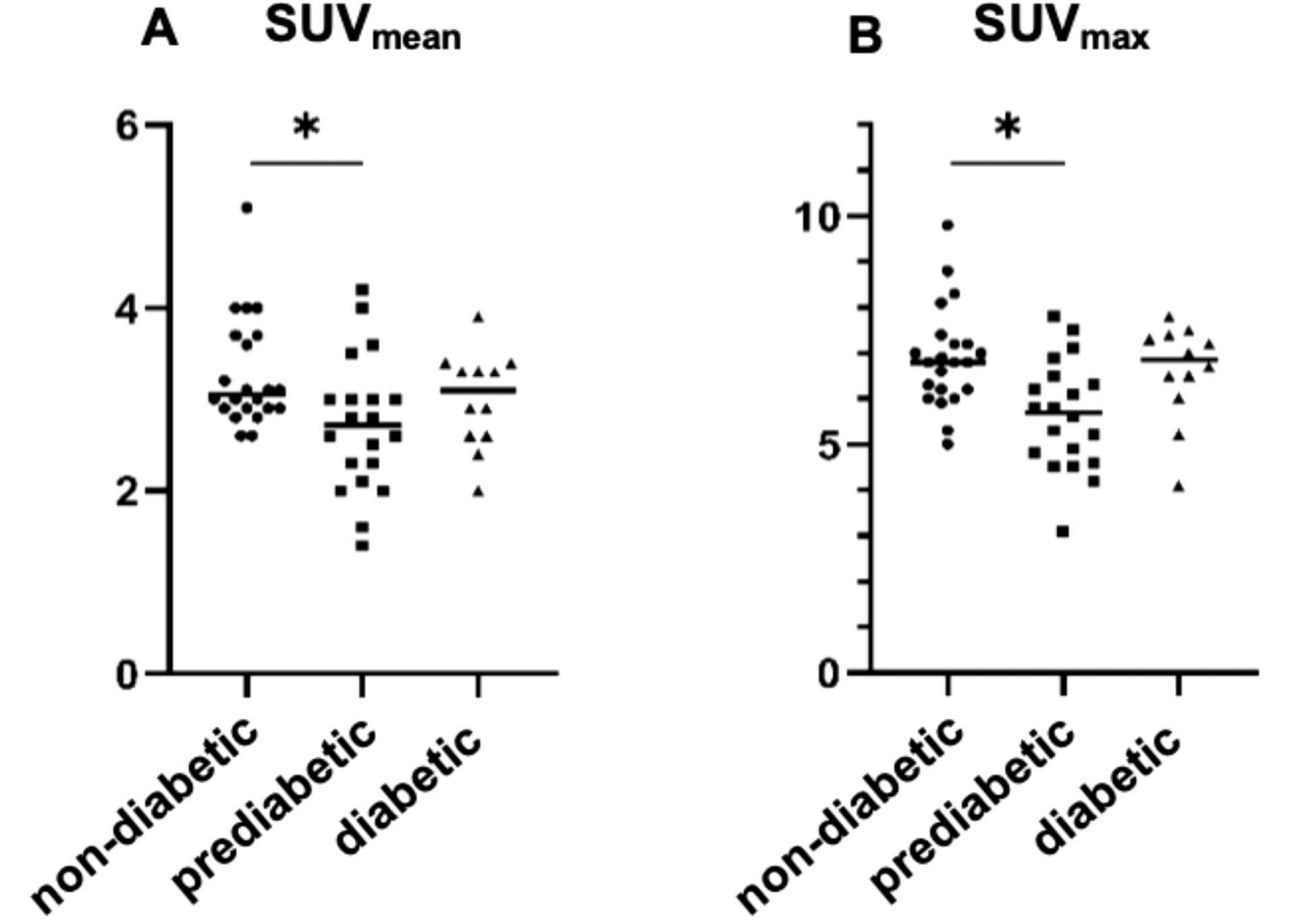
Comparison of SSTR expression in patients with normal glucose homeostasis, impaired glucose homeostasis and with diabetes mellitus. SSTR expression on [^68^Ga]Ga-DOTATOC-PET/CT was significantly decreased in prediabetic patients (HbA_1c_: 5.7 - 6.4%, SUV_max_: 5.7 (Q1: 4.8, Q3: 6.4) SUV_mean_ **(A)**: 2.7 (Q1: 2.3, Q3: 3.0)) compared to non-diabetic patients (HbA_1c_: 4.0 - 5.6%; SUV_max_ **(B)**: 6.8 (Q1: 6.2, Q3: 7.2); SUV_mean_: 3.1 (Q1: 2.9, Q3: 3.7); p = 0.004 and p = 0.033). There was no significant difference in patients with diabetes mellitus (intake of diabetes medication and/or HbA_1c_ ≥ 6.5%) and patients with normal glucose homeostasis or prediabetic patients.

Tracer uptake was significantly lower in patients with prediabetes compared to patients with normal glucose homeostasis SUV_max_ (p = 0.009) and SUV_mean_ (p = 0.033). There was no significant difference in uptake between patients with T2D and patients with normal glucose homeostasis. A trend was observed for elevated SUV_max_ in patients with T2D and antidiabetic medication compared to patients with prediabetes which did not reach statistical significance (**Figure 3**).

The effect of antidiabetic medication was then investigated for the whole cohort.

**Figure 4:**
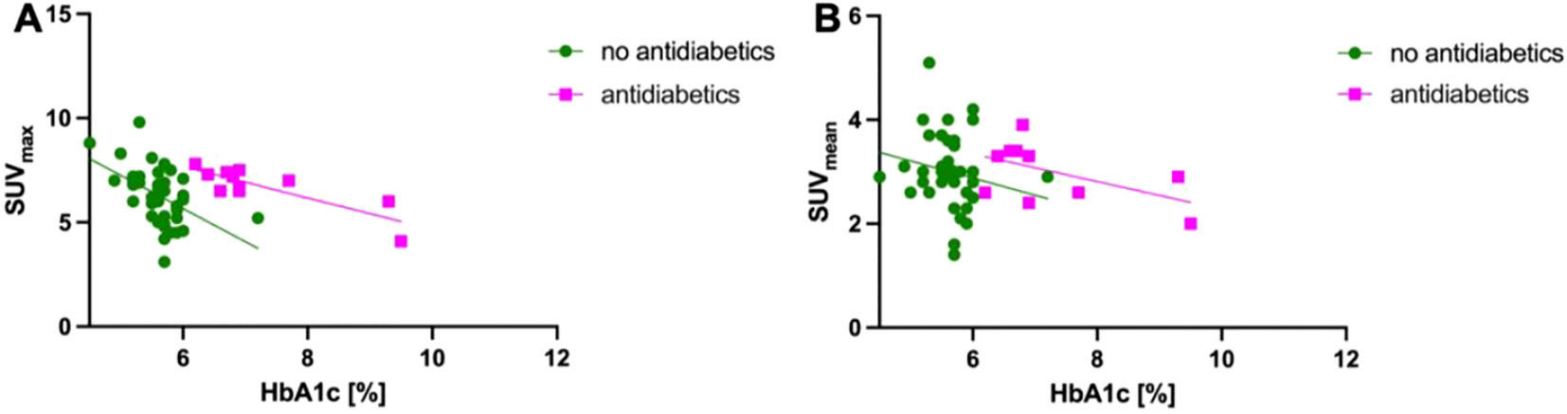
Correlation of HbA_1c_ and SUV. **(A)** Correlation analysis showed a significant correlation between HbA_1c_ and SUV_max_ (r^2^ = 0.267; p < 0.001) in patients without antidiabetic medication as well as a in patients with antidiabetic medication (r^2^ = 0.450; p = 0.027). **(B)** Correlation analysis showed a significant correlation between HbA_1c_ and SUV_mean_ (r^2^ = 0.094; p = 0.046) in patients without antidiabetic medication but not in patients with antidiabetic medication (r^2^ = 0.239; p = 0.130).

Correlation analysis showed a significant correlation between HbA_1c_ and SUV_max_ (r^2^ = 0.267; p < 0.001) or SUV_mean_ (r^2^ = 0.094; p = 0.046) in all patients without antidiabetic medication at the PET imaging and a significant correlation between HbA_1c_ and SUV_max_ in patients with antidiabetic medication (r^2^ = 0.450; p = 0.027) (**Figure 4**).

Additionally, the multiple linear regression analysis correcting for the intake of antidiabetic drugs revealed a significant association between HbA_1c_ and SUV_max_ (r^2^ = 0.667; p < 0.001). The confidence intervals were -0.371 to -0.135 (p < 0.001) for the independent variable HbA_1c_ and 1.393 to 2.120 (p < 0.001) for the covariate intake of antidiabetic drugs. There was a trend for association between the HbA_1c_ and the SUV_mean_ that did not reach statistical significance (p = 0.061). Use of antidiabetic drugs was confirmed as a significant covariable (r^2^ = 0.577; CI 1.236 to 2.047; p < 0.001) (**Table 2**).

**Table 2:** Multiple linear regression: Influence of uptake values and clinical characteristics on HbA_1c_.

Independent variables	R-squared	95% Confidence interval		P-Value
		Lower	Upper	
SUV_max_	0.667	-0.371	-0.135	<0.001
Antidiabetic medication		1.393	2.120	<0.001
SUV_mean_	0.577	-0.475	0.011	0.061
Antidiabetic medication		1.236	2.047	<0.001

## Discussion

In this pilot study, we observed a significant reduction in tracer uptake of the pancreas on [^68^Ga]Ga-DOTATOC-PET/CT in prediabetic patients (HbA_1c_ 5.7 - 6.4%) compared to those with normal glucose homeostasis (HbA_1c_ < 5.7%). Conversely, the use of antidiabetic medication was associated with increased pancreatic tracer uptake on [^68^Ga]Ga-DOTATOC-PET/CT. Our findings suggest that pancreatic radioligand uptake on [^68^Ga]Ga-DOTATOC-PET/CT is sensitive to fluctuations in glucose homeostasis and to antidiabetic medication.

Our findings show reduced pancreatic uptake on [^68^Ga]Ga-DOTATOC-PET/CT in patients with a glucose metabolism disorder aligning with prior findings of decreased membrane SST2 immunoreactivity observed in pancreatic islets from human donors with T2D (23). In prior studies, it was also shown, that T2D was associated with reduced β cell mass and function, lower δ cell counts and diminished somatostatin secretion (24–27). Previous molecular imaging studies on SST radioligand tracer uptake and glucose metabolism gave inconsistent results: A small study including four patients with diabetes found no significant impact of diabetes on the ^99m^Tc-HYNIC-TOC uptake in single-photon emission computed tomography/CT (SPECT/CT) (28). However, this study was limited by its small sample size and the lower image resolution of SPECT compared to PET (28–30). In contrast, another study using [^68^Ga]Ga-DOTATOC-PET/CT reported a significant correlation between tracer uptake in the pancreatic *processus uncinatus* and blood glucose levels, aligning with our findings (20). Previous studies have also discussed an association between pancreatic radioligand uptake and pancreatic polypeptide (PP) cell hyperplasia, especially prevalent in the *processus uncinatus* where these cells constitute 55-90% of islet cell volume (13, 31). PP cell deficiency has been linked to glucose intolerance and insulin resistance (32), and since PP cells express SST2, reduced radioligand uptake, may, in part, reflect PP cell deficiency.

We observed a positive association of antidiabetic medication and radioligand uptake in the pancreas. Consistent with our results, a previous study pointed out, that the pancreatic uptake was increased more often in patients taking antidiabetic medication compared to patients without antidiabetic drugs (13). In another previous study on pituitary *SSTR* mRNA expression, diabetic rats showed significantly reduced *SSTR5* mRNA expression levels compared to non-diabetic controls. After the administration of insulin therapy the *SSTR5* mRNA expression in the diabetic rats was comparable to the *SSTR5* mRNA expression of non-diabetic controls (33). These results seem to align with our findings which showed decreased radioligand uptake of the pancreas in patients with prediabetes, while diabetic patients receiving antidiabetic medication exhibit pancreatic radioligand uptake levels comparable to those of patients with normal glucose homeostasis. Upregulation of pituitary SST expression has been observed not only with insulin therapy but also with biguanides like metformin, where upregulated *SST2* and *SST5* expression in primate pituitaries has been shown *in vitro* (34). For several antidiabetic drugs (e.g. DPP4-agonists), a stimulation of somatostatin secretion has been reported (35). However, little is known about the effect of antidiabetic drugs on the pancreatic SST expression.

Our data reveal a significant negative correlation of SUV_max_ with HbA_1c_, while multiple regression analysis with SUV_mean_ only showed a trend towards significance. The weaker correlation for SUV_mean_ may be attributed to a partial volume effect, given the inhomogeneous distribution of islet cells, which constitute only about 2% of the pancreatic volume (36–41). Additionally, SUV_mean_ is susceptible to minor inaccuracies in volumetric delineation of the pancreas, whereas SUV_max_ is less impacted.

Pancreatic radioligand uptake on SSTR-PET, particularly in the *processus uncinatus*, is a known challenge in diagnosing pancreatic neuroendocrine tumors (13, 42). Individual variations in non-oncologic somatostatin receptor expression of the pancreas might affect the sensitivity and the diagnostic certainty when investigating [^68^Ga]Ga-DOTATOC-PET/CT scans in patients with pancreatic neuroendocrine tumors. Therefore, it might be necessary to consider the individual glucose homeostasis of patients undergoing SSTR-PET/CT analogous to the fasting blood glucose level of patients undergoing FDG-PET/CT (43).

A primary limitation of this study is that the description of diabetic and prediabetic status was determined solely on HbA_1c_ measurements, an indirect measure of long-term glucose control rather than a direct assessment of β cell activity. Additionally, the HbA_1c_ might be influenced by factors such as altered erythrocyte turnover and acute glycemic fluctuations (44, 45). It should be borne in mind, however, that this study was merely a retrospective pilot project involving patients of the outpatient Department of Nuclear Medicine, most of whom were referred from private medical practices. The HbA_1c_ was the only consistently documented marker of glucose metabolism across all included patients. Consequently, the absence of additional markers, including fasting glucose, insulin, and HOMA index, represents a limitation in the precise characterization of glucose metabolism disorders.

Furthermore, the limited size of the study cohort may have had a bearing on the analysis of the data and the interpretation of the findings. All patients who met the inclusion criteria and underwent [^68^Ga]Ga-DOTATOC-PET/CT at the institution were included in this study, ensuring a comprehensive assessment within the available clinical dataset. However, due to a transition in clinical practice to [^18^F]SiTATE, further expansion of this retrospective cohort is not feasible. Nevertheless, the findings provide important preliminary insights into the interplay between glucose metabolism disorders and somatostatin receptor-targeted imaging.

To address this limitation of this pilot study, a prospective study utilizing [^18^F]SiTATE is currently underway, incorporating a more extensive endocrinological assessment, including insulin, C-peptide, and additional metabolic parameters. This approach is expected to enable a more refined evaluation of glucose metabolism and its potential impact on somatostatin receptor-targeted imaging. The present findings should therefore be regarded as hypothesis-generating, providing a foundation for future investigations that will facilitate a more differentiated interpretation of metabolic influences on radiotracer uptake.

It is additionally recognized, that further factors may influence pancreatic function and glucose metabolism as well as SST expression and pancreatic uptake, such as dietary habits, body mass index, and comorbidities. However, due to the retrospective design of the study and its conduct within a diagnostic imaging department, these parameters were not systematically collected, thereby representing a limitation in the analysis. Acknowledging the importance of these factors, it is proposed that future studies incorporate a structured questionnaire in collaboration with endocrinology specialists to comprehensively assess the impact of lifestyle and metabolic factors on SST expression.

In this study, patients were treated with a range of antidiabetic agents, including metformin, insulin, and DPP4 inhibitors, often in combination, however, the impact of antidiabetic medications on tracer uptake is a pivotal factor in the interpretation of PET imaging (35). Due to the small sample size and the use of multimodal therapies, precluded the ability to discern the distinct contributions of each medication on tracer uptake, however, this must be considered as a potential confounding factor when interpreting the results. Consequently, sturdies with controlled cohorts are required to systematically investigate the impact of various antidiabetic treatments on SST expression and PET tracer uptake.

In conclusion, the present study identified a significant negative association between pancreatic [^68^Ga]Ga-DOTATOC-uptake and HbA_1c_. It also shows that antidiabetic treatment increases tracer uptake. This may have a clinical impact on the sensitivity of SSTR-PET in the diagnosis of pancreatic NETs as well as on PET reading in patients with diabetes. As this was a retrospective pilot study, these findings emphasize the necessity for further research utilizing larger, prospectively collected datasets that encompass comprehensive metabolic profiling. The results of this study provide a foundation for future studies and contribute to a deeper understanding of the metabolic influences on somatostatin receptor-targeted imaging.

## Data Availability

The raw data supporting the conclusions of this article will be made available by the authors, without undue reservation.

## Author’s note

Datasets of this publication were presented at the Deutsche Diabetes Kongress 2023 (Diabetes Congress of the German Diabetes Society) and the SNMMI Annual Conference 2023.
